# Data-driven information for action

**DOI:** 10.1007/s11612-023-00666-9

**Published:** 2023-02-21

**Authors:** Kristin Wulff, Hanne Finnestrand

**Affiliations:** 1 Faculty of Economics and Management, Department of Industrial Economics and Technology Management, Norwegian University of Science and Technology, Trondheim, Norway; 2 Kantega AS, Trondheim, Norway

**Keywords:** Data-driven, Machine learning, Sociotechnical systems design, Organizational design, Information for action, Datengesteurt, Maschinelles Lernen, Soziotechnische Systemgestaltung, Organisationsdesign, Information zum Handeln

## Abstract

Because of the increase in data and the possibilities created by machine learning, organizations are now looking to become more data-driven. In sociotechnical systems design there has been a focus on designing information for action to support decentralized organizations. The purpose of this article, published in Gruppe. Interaktion. Organisation. is to discuss how data may be gathered and used in organizations striving to become data-driven.

Explorations are based on interviews with experts (leaders and designers) in 13 organizations working on becoming more data-driven.

This study points to 4 findings: first, if someone is expected to record data that informs other people’s actions can lead to data quality issues, which can be mitigated by providing transparency or supporting a *joint information for action *as an organizational design choice. Second, as organizations are becoming more data-driven, many tasks performed in the organization become design-related. This influences the type of data recorded and used for action. Third, more of the people in the organizations engage in designing the information for action for themselves and others, which means that they might need reskilling. Fourth, the boundaries of what can be considered information for action and for whom should by explored and reflected upon by the people involved in the (re)design.

This means that, as organizations strive to become data-driven, the sociotechnical principle of information flow becomes a central challenge. To ensure quality organizations, there is a need to upskill or reskill employees so that they are able to design and use data for action.

## Abstract (de)

Aufgrund der Zunahme von Daten und der Möglichkeiten, die Maschinelles Lernen schafft, versuchen Organisationen nun mehr und mehr datengesteuert zu werden. Um dezentralisierte Organisation(en) zu unterstützen, hat soziotechnische Systemgestaltung den Schwerpunkt auf die Gestaltung von Information zum Handeln gelegt. Der Zweck dieses Artikels, veröffentlicht in Gruppe. Interaktion. Organisation., ist zu erörtern, wie Organisationen, die bestrebt sind, datengesteuert zu werden, Daten sammeln und verwenden können.

Die Untersuchungen basieren auf Interviews mit Experten (Führungskräften und Designern) in 13 Organisationen, die an vermehrter Datensteuerung arbeiten.

Die Studie weist auf vier Erkenntnisse hin: Erstens, wenn von jemandem erwartet wird, Daten zu erfassen, die das Handeln von Anderen beeinflussen, kann dies zu Datenqualitätsproblemen führen, die durch eine Gestaltungswahl von Transparenz und der Unterstützung von *gemeinsamer Information zum Handeln* abgemildert werden können. Zweitens, im Laufe vermehrter Datensteuerung werden die in Organisationen ausgeführten Aufgaben zunehmend gestaltungsbezogen. Dies beeinflusst die Art der erfassten und zum Handeln verwendeten Daten. Drittens, da mehr Beschäftigte Information zum Handeln für sich selbst und andere gestalten, müssen diese möglicherweise weitergebildet werden. Viertens, die Grenzen dessen, was als Information zum Handeln angesehen wird und für wen, sollte von den Beschäftigten die an der (Neu‑)Gestaltung beteiligt sind sondiert und überdacht werden.

Das bedeutet, wenn Organisationen danach streben, datengesteuert zu werden, wird der soziotechnische Ansatz zum Informationsfluss zur zentralen Herausforderung. Um die Qualität in und von Organisationen zu gewährleisten, müssen Mitarbeiter weitergebildet oder umgeschult werden, um Daten zum Handeln gestalten und nutzen zu können.

## Introduction and theoretical positioning

Customer expectation around fast delivery and personalization of services is increasing. Insurance payouts are often received seconds after entering the insurance claim, and we expect to receive fast feedback when applying for a car loan. The possibilities to personalize and automatize are based on the increase in stored data because of the digitization of data and digitalization of work (Gobble 2018). Such data, paired with the possibilities of Machine Learning (ML), has created all kinds of iconic personalized services, from the Netflix Recommendation Engine to picture recognition systems that can read the label of wine bottles in photos. According to Brynjolfsson and McAfee (2014), organizations are facing the largest transformation since the industrial revolution. Digital transformation will alter expertise, change work design and boundaries, and enable new ways to coordinate and control (Faraj et al. 2018). An essential part of the digital transformation is to use data for predictions and to prescribe action (Iansiti and Lakhani 2020). This can be referred to as data-driven decision-making (Brynjolfsson and McElheran 2016), and/or becoming a *data-driven organization* (Berndtsson et al. 2018). When redesigning such organizations it is vital to use design guidelines that result in quality work and a flexible organization.

When digital technology is used to change the sociotechnical structures, it can be defined as *digitalization*. A *digital transformation* happens when the digitalization and/or digital innovation over time transforms how business is conducted and this transformation changes an entire organization or industry (Osmundsen et al. 2018). To provide guidelines for digital transformations, sociotechnical systems design (STSD) with its design principles and parameters can be used (Babüroğlu and Selsky 2021; Pasmore et al. 2019) to improve the quality of work (de Sitter et al. 1997; Van Eijnatten 1993; Vriens and Achterbergh 2011). STSD can also inform the IT architecture, as seen in the description by Govers and van Amelsvoort (2018) where processes are parallelized and supported with one IT information interface each, in what they call Archipelago architecture. One important design principle advocated by Cherns (1987), and reinforced by others, is access to information for action. For the people in the organization who need it in order to carry out their work and to handle the variance they experience it is crucial that the information reaches them.

Organizations have always used data to help them make decision, but what is new is the increased volume and variety of data, the possibility to share the data, and its use in near real-time (Bean 2021). To be able to use the data in innovative ways, organizational learning is crucial (Mikalef and Krogstie 2020). The data is explored to find ways of increasing the competitive advantage of an organization (Provost and Fawcett 2013). Till now, the use have been mainly for the existing business (Brock and von Wangenheim 2019) to improve customer experiences, create new products and services, make better decisions, make the work processes more efficient, and reduce costs (Benbya et al. 2020). Data have already inspired new business models, and more is expected to in the years to come (Teece and Linden 2017). The improvements in decision-making because of an increased use of data can be seen on strategic, tactical and operational decisions (Halper and Stodder 2017).

The problems in creating a data-driven organization are mainly non-technical, for instance whether to use data to take decisive action or whether data is trusted (Halper and Stodder 2017). The data quality is also often a challenge (Janssen et al. 2017). To improve the organizational use of data, the suggestion is to increase data literacy in the organization (Berndtsson and Svahn 2020). However, there is also a need to be able to use the data to improve the way of working. This can be achieved by testing alternative response patterns and using data regarding outcome, thus selecting the most appropriate way of working (Herbst 1974).

Unfortunately, the fast and potentially very profitable technological innovations may tempt organizations to take shortcuts (Zuboff 2019) and/or to choose to have a less conscious relationship with how a data-driven organization can help build quality of work. Building a good quality work system is therefore a design choice. Sociotechnical systems design (STSD) theory has been reintroduced as a possible way to understand and design the digital transformation (Babüroğlu and Selsky 2021; Pasmore et al. 2019). Makarius et al. (2020) describes how STS can aid in designing the different roles that humans can fill vis-à-vis AI, for instance, controller, collaborator, or conductor. Claussen et al. (2019) suggests that the digitalization calls for “STS-beyond” where the paradox of power-related control versus trust-based control is included as a deliberation in the design of technology. Haga (2019) illustrates how the deliberations and dialogue described by Pava are a beneficial way for the organization to create participative processes when designing and redesigning technology (Pava 1983, 1986). Central to STSD is the work unit’s ability and autonomy to deal with the variance it experiences. This is necessary to ensure quality of work and an increased innovation potential (de Sitter et al. 1997; Vriens and Achterbergh 2011). From work in the coal mines (Trist 1981; Trist and Bamforth 1951) to software development (Nerur et al. 2010), the variance caused by unpredictability of the context and content impacts how the work of an organization should be designed (Boonstra and Reezigt 2019). STS designs are aimed at improving the quality of work and organization through adaption of contents and integration of technology and human tasks (Van Eijnatten 1993). In order to succeed, humans need access to information (Cherns 1987).

To enable the people in the organization to make decisions and take action based on data, the data must be put into a relevant context (Davenport et al. 2012). It must become information and reach the people that can use it, in a decentralized organizational design (Brödner and Latniak 2003). Cherns (1976) states that the information should be designed so that the information flows to the people who need it to do their job, and not to managers or others who might use it for control. In a later article, Cherns (1987) specifies more clearly the three uses of information. Information can be used for *control,* it can be used for *record,* and it can be used for *action*. Control is about using information to assert power, record is for showing what has happened and is happening, while information for action means that those who are required to act get the information they need. The principle of information flow is used and further developed by several authors. For instance Curşeu et al. (2021) discuss how unequal power may be balanced with information flow, and Lin and Cornford (2000) to explain the connection between information flow and variance. Information for action is used by, for instance, Knight and Parker (2021) who refer to it with regards to the need for timely feedback to the persons doing the job, and Meacham (2022) who uses it when researching fire safety. Information for control can be seen in what is called Algorithmic Management, where algorithms, for instance, monitor and evaluate processes, for instance the performance of workers. A mediating effect when using Algorithmic management, inspired by STSD, is transparency, perceived fairness and the worker’s possibility to control the system (Parent-Rocheleau and Parker 2022).

Handling the variance of the market is seen by many as a managerial task (Emery and Thorsrud 1976), and with the increase in data comes the possibility to use insights delivered by business analytics to support managerial decision-making (Delen and Ram 2018). Following this perspective, the managers in an organization would expect the information for action to be handed to them. This view contradicts the design parameters and principles of STSD, which gives the information for action to the people doing the work. Designing the control system bottom-up is a vital part of the STSD, for instance, the STSD by de Sitter et al. (1997). The idea that management make all the decisions in the organization is truly challenged when data becomes widely available and this leads to a shift in power from management to for instance, analytics experts (Galbraith 2014). By combining the data into data products that can be made available to all, the possibility to share real-time data both internally and externally increases (Vidgen et al. 2017). This means that information that managers may have considered to be theirs, may now be given to other people in the organization, and also to people outside the organization, for instance, startups. As Mitki et al. (2019) demonstrated, this sharing of “managerial” information to workers can be very beneficial for the development of the company. The access to information is central in turbulent environments (Govers and Südmeier 2016), and by providing such information one can develop a learning organization (Herbst 1974, 1993). That is, an organization that is able to use data to perform double-loop learning (Argyris and Schon 1974) where the people doing the work is informed in a way that gives them the opportunity to try new ways responding. Still, it may be difficult for managers to relinquish control and give the decision-making responsibility based on data to workers. Therefore, this is an area that will benefit from research into the transition around information gathering and use changes.

This leads to the following question: *how may data be gathered and used for information when organizations strive to become data-driven?*

Norway ranks fifth on the European Digital Economy and Society Index (DESI), behind Finland, Denmark, the Netherlands and Sweden. The DESI index measures the level of a country’s digitalization. The finance and public sectors are two front runners in the area of digitalization. The public sector has been inspired by the governmental strategy of “one digital public sector” (Norwegian Government 2019) while finance is leading the way in becoming more data-driven (Almquist et al. 2021), assisted by the fact that a large part of their offerings can be produced and delivered digitally. In this study, we have interviewed experts and leaders from organizations becoming data-driven in both sectors. Their organizations are on different stages of becoming data-driven: some are only just starting out, others have been trying to transform, but without much success, while the third kind are ahead of the general development and already reaping huge benefits from their data. In this explorative study, we have collected viewpoints from the experts on the state of the development in their organization through retrospective interviews.

## Research design

The empirical data for this article is based on a research project conducted by a group of nine data professionals and leaders, including the first author, from a software consultancy. Because the research we had found was all international, we decided that it would be important to get a better grip on the situation in Norway. This means that our sampling strategy was purposive (Miles et al. 2018). The purpose we followed when sampling was to find the people that were most experienced in the area of data and ML use. The AI research group collaboratively designed the inquiry (Shani and Coghlan 2021). We carried out interviews on topics related to data and ML with experts mainly from the banking and finance and public sectors—finance because it is one of the leading sectors (Almquist et al. 2021), and the public sector because of the government’s requirements to use and share data (Norwegian Government 2019). We interviewed both front runners and organizations that were at the beginning of their journey.

We conducted a total of 24 expert interviews across 13 organizations in the period between May 2020 and March 2021 (Table 1). The interview guide for the qualitative, semi-structured interviews was iteratively and collaboratively developed by the group doing the interviews (Holstein and Gubrium 1995). Because we wanted to have rich context descriptions, we decided to have little prior instrumentation (Miles et al. 2018). The interviews were open-ended and explorative (Gudmundsdottir 1996) and we built on the knowledge we gathered and changed the next interview accordingly.

The main focus of the questions was to understand how top management was involved, what roles were defined in top management and other levels of the organization, how the ML work was organized, how the interaction between the design team and the rest of the organization was handled, what experiences and challenges the interviewees meant that their organization had had with regards to becoming more data-driven, and what areas the interviewees saw as having benefitted from using ML.

**Table 1:** Overview of the organizations and interviewees in the study

Nr.	Role	Organization	Approx. org. size	Sector	Type of business
1	Development Director	Alvarinho	100	Private	Software products
2	Senior Vice President (VP)	Barbera	9000	Private	Finance
3	Head of Robotics & AI	Barbera	9000	Private	Finance
4	Chief Data & Analytics Officer (CDAO)	Barbera	9000	Private	Finance
5	Programme Leader Anti-Money Laundering (AML)	Carignan	7500	Private	Finance
6	IT Developer	Carignan	7500	Private	Finance
7	Chief Information Officer	Dolcetto	400	Public	Directorate
8	Credit Manager	Egiodola	50	Private	Finance
9	Solution Architect	Egiodola	50	Private	Finance
10	Head of Innovation	Egiodola	50	Private	Finance
11	Chief Data Officer (CDO)	Fiano	900	Private	Finance
12	Head of Automation and Business Analytics	Fiano	900	Private	Finance
13	Head of Machine Learning and AI	Fiano	900	Private	Finance
14	Head of Architecture and Platform	Garnacha	250	Private	Finance
15	Product Owner Data Platform	Hamashara	6600	Public	Directorate
16	Head of Automation	Hamashara	6600	Public	Directorate
17	Director	Impigno	2600	Private	Finance
18	Senior Vice President IT	Impigno	2600	Private	Finance
19	Senior Enterprise Architect	Impigno	2600	Private	Finance
20	Analysis Leader	Jampal	550	Public	Energy provider
21	Chief Technology Officer	Katsano	1250	Private	Finance
22	Strategic Advisor	Lambrusca	1500	Private	Finance
23	Executive Director	Lambrusca	1500	Private	Finance
24	Digital Product Manager	Marsanne	550	Private	Maritime technology

Of the 24 interviews, 22 were audio recorded. The recording was done by connecting an audio recorder to the MS Teams meeting. The transcription was done verbatim, i.e., everything that was said, including “ehms” and such, was written down (Poland 1995), without noting any nonverbal communication. Two of the interviewees were Swedish, and those were translated directly to Norwegian in the transcription, while the two English interviews were transcribed in English. Once the transcription was approved, the audio recording was deleted. Due to COVID-19, 23 of the 24 interviews were conducted online on MS Teams. The questions and a consent form were approved by the Norwegian Centre for Research Data (NSD).

A thorough first cycle coding was performed as the transcribed interviews were coded in Quirkos with the use of in vivo codes, concept codes and process codes (Miles et al. 2018). The interviews were analyzed using theme-based analysis with a focus on the content of the narrative, as opposed to a structural analysis focusing on how the story is told (Riessman 2008). The coding was validated by two representatives from the research group, contributing to the confirmability of the selection to ascertain that the logic was coherent, in what Lincoln and Guba (1985) call a confirmability audit. They suggested a two-level structure, which was implemented by the first author. Then, the authors agreed on which codes to research further, thus engaging in an inductive-deductive process. To get an overview of the quotes in the different codes, the first author printed, cut out, and sorted the quotes and divided those codes that had become too broad into subcodes (Miles et al. 2018) before entering them into a mindmap. The mindmap was then studied by the first author and the research group, and patterns of positive and negative influence pointed out and discussed. These were described in causal fragments, before joining them into two causal networks (Miles et al. 2018). One causal network depicted the actions taken by organizations that were very successful in their work to become more data-driven, and the other the actions taken by the less advanced organizations. The causal networks were tested on three colleagues outside the AI research group in a peer debrief (Lincoln and Guba 2016). These discussions and visualizations provided a foundation for selecting an area of difference between the organizations and helped the authors define the area of analysis. The selected area was information flow and its three variants: information for record, action, and control. Thereafter, the selected codes (quirks) and quotes were analyzed to find illustrative examples to present here. The quotes in Norwegian were translated by the first author and double-checked by the second author.

According to Bulling (2018), organizational change can be described by its degree of change, how broad the employee involvement is, and who leads the change. The AI research group used this categorization to evaluate the organizations. In addition to the three factors described by Bulling, we have added information from the interviews on when the organizations started using ML actively (that is with the intention for using it in production) and whether any of their solutions had reached production at the time of the interview.

As can be seen from Table 2, most of the organizations’ degree of organizational change so far had been incremental, for instance by creating ML models to detect fraud or reduce customer churn. This can also be seen from the different examples we will present next. In the following we have chosen to focus our analysis on two of the three variants of information flow: information for record and for action. This is partly because the third variant, information for control, is something Cherns (1987) advises against, and partly because we believe that control issues will have to be researched in another way, for instance, by observing how the information flow is actually designed. We start our exploration with the first contact with the data—the data gathering.

**Table 2:** Evaluation with regards to becoming data-driven based on Bulling (2018) and status given in the interviews

	Degree of organizational change (Bulling 2018)			Status in the organizations	
Organization	Degree of change	Degree of involvement	Who is leading the change	Active use of ML since	Solutions in production
*Alvarinho*	Incremental	Several groups	Strategic management	2018	Yes
*Barbera*	Incremental	The whole organization	Strategic management	2019	Yes
*Carignan*	Incremental	One team	The developers	2018	Yes
*Dolcetto*	Incremental	Single persons	Middle management	2020	No
*Egiodola*	Incremental	Single persons	Middle management	2013	No
*Fiano*	Incremental	Several groups	Strategic management	2018	Yes
*Garnacha*	Incremental	One team	Middle management	2020	No
*Hamashara*	Incremental	Single persons + one team	The analysts and developers	2019	Yes
*Impigno*	Incremental	One team	Strategic management	2017	Yes
*Jampal*	Incremental	One team	Analysts	2017	Yes
*Katsano*	Incremental	Several groups	Strategic management	2016	Yes
*Lambrusca*	Incremental	Single persons	Strategic management	2020	No
*Marsanne*	Radical	Several groups	Business and digital developers	2016	Yes

## The empirical perspective—what the experts say

### Data gathering

The informants were aware of the new possibilities for designing information that emerges from recording data through digitalization. However, the organizational awareness differed. Some of them were particularly conscious about what to store and what not, while others struggled with getting data recorded in a retrievable way.

Now, we are much more aware on how we gather data and store it from all kinds of customer processes and business processes. That gives us a totally different set of data than we had earlier. Before, you had to access many different sources to find and gather the data. It is still a challenge to group all the data before one begins the processing, but much of the data is already structured so that it is easy to process without needing to adapt it to for instance a machine learning model. (Senior VP, Barbera)

In this quote from Barbera, we can see how important it is that the data is easily accessible, for example for building predictive ML models. Barbera reported that they had been quite conscious and explicit about decisions on what to record from the different processes. However, other organizations had not reflected about it in the same way. They were not digitalized to the same degree, and digitalization was hampered by differences in how the data was recorded. For instance, those organizations realized that people recorded data for their own actions only, or that they recorded data without knowing what it was used for and therefore stopped recording it at some point. This is illustrated in the following quote:

Since we have a very low awareness on what we can get from the data, we have not been good at taking care of our data. We still write information that is important for operations down by hand and store it in ring binders in people’s offices. It is incredible. For the operation of the power plants for instance, there has been no trend analytics [predictive analytics] on any of them. Here, data was noted in books, and at some point, they just stopped recording it. And no-one restartet doing it, because they didn’t know what to do with it. (Analysis leader, Jampal)

The fact that someone stops recording the data because they do not know what to do with it, points to a process where there is no focus on information for action. The data that was recorded in binders may have been information for action at one point, but storing it in binders is not consistent with using the data for learning purposes as it is hard to compare trends and do other analyses on such data.

How to store data was seen by some as a personal choice, and others’ need for the same data was not seen or acknowledged. Some informants told us about professionals who regarded their data and models as their personal intellectual property, as illustrated in this quote:

You have some professional areas that can be considered half art and half engineering. For instance, detecting quick clay through taking drill core samples. Then you get a pressure profile and can measure water penetration and guess whether there is quick clay present or not. And this is not an exact science, it is intuition and such. The mathematical models that people use are not efficient, it is 50/50. It seems that people saw this as their interpreations, it was their intellectual property. So, when we began doing machine learning on this many protested against sharing their data, but after a while we gathered enough data to run predictions and found 97% correlation between the drill core samples and the more precise method of core drilling. (Head of Automation, Hamashara)

By calling it half art/half engineering, the informant illustrates how the ownership of the data is important to each individual. This makes it understandable that it is hard to share the data. Therefore, it seems important to find ways of increasing individual awareness that the data gathered can be used for collective learning purposes.

### Data use

All of the informants were passionate about using the data available to inform action, as illustrated in the following quote:

What I want is that it [information] should give us better insight. In a way it should add brainpower to the people analyzing data and help us to see patterns in data to pick up changes early. For instance, changes in customer behavior or other things, changes that happens around other actors. We see it as an added processing power that is connected, that fits what people are looking for themselves already. (Solution architect, Egiodola)

However, the passion of the informants sometimes turned into frustration when they met road blocks in the organizational design that reduced the organizations ability to make use of the data.

I believe this is an awareness thing. If you look at the value chain, the “data gang” or the people having the data mindset sit at the end of the value chain. If you want to get the optimal setting you need to move that mindset to the front of the value chain where the customer is, and the transaction occurs. This could lead to an awareness for the people on the front desk to understand that all the information about the customer or the process has great value later in the process, and that the quality of this data needs to be perfect. Then I think we can succeed. But it will take time. What many companies do is that they improve the low data quality afterwards. That is not a good choice, you need to be able to improve the front of the value chain. (Senior VP, Barbera)

One informant pointed out that information which was built for use in the ML model was discovered to have other uses for action as well. For example, one of the organizations discovered that the data that was fed to the ML model for identifying any possible anti-money-laundering cases was so valuable in itself, that they chose to make it available to the people working with such cases.

We began with a simple supervised ML model. We realized then that the data we use to train the model gives a lot of value to the banks as it is. There is a lot of data that is relevant when you are working on anti-money-laundering cases, but that the case workers have not had access to. So, in addition to the ML project we have built and made available a report with the same data. (Program Leader AML, Carignan)

Here, the expectations of what the data would be used for expanded, which means that one cannot always determine the usefulness of data in advance. In another case, the organization discovered that they wanted data that they did not have. Because of COVID-19, some organizations experienced an unprecedented increase in uncertainty. For the leaders of these organizations, there was an increased need for information to give insight into possible strategic actions. For instance, a board of directors was mentioned as a group needing more information to handle the situation.

We have an interesting case now, because corona has done things with our business that is impossible to simulate. For instance, at the worst time, our revenue halved. So, now we have a board that jumps up and down and wonders, ‘We must have estimates, what will this mean for the result this year, and what will it mean for next year?’ They think we are fortune tellers. We do not know what will happen. Will people be able to afford to pay the same as before? (Solution Architect, Egiodola)

The situation that the board of directors—and many others—were in, illustrates the limits of being data-driven. Without data from similar situations, it can be impossible to do predictions. Therefore, to have data that shows what is happening in real-time can be beneficial. This was explained to us by two of our informants. These two organizations shared data with the Norwegian government and Statistics Norway to support decisions in connection with COVID-19.

Many governmental actors like for instance Statistics Norway use data from the payment infrastructure in their models to increase the quality of macro-economical decisions. Delivering such data is part of our permission to run this infrastructure. One example where the insight from the infrastructure has been used for big and fundamental decisions for Norway was when a committee evaluated the work done in connection with COVID-19 and recommended how to proceed. In such macro-decisions, the possibility to have easy access, continuously and preferably in real-time, is essential (Director, Impigno)

This means that the necessary data may be shared both inside and between organizations.

### Data sharing

Public organizations in Norway are required to share data outside their organization (data.norge.no). This led one of the public organizations to state that *“data that is not exposed is not real” (Head of Automation, Hamashara).* To expose data is to make it available for others to extract. Another public organization (Dolcetto) mentioned how they shared data with public organizations for real-time use of the data in those organizations’ software solutions. In this way they contribute to better services from different public organizations, and probably also better coordination. One informant told us about a gas station chain that asked for access to traffic pattern data to better estimate the number of buns to warm up, thereby reducing the waste of food. In this way, the data was intended to support another organization’s actions. The data shared with government was to provide the foundation for decisions that had to be made, which function as information for action. Private organizations also shared data, both internally between business areas (Marsanne) and externally: one of the banks asked the customers’ consent for using their transaction data to inspire them to reduce their CO2 footprint.

Some of the informants explained how access to data had been a discussion point in their organization. Some organizations had previously had a strict governance framework for the data, partly because of legal regulations and partly for security reasons. This was challenged due to new data platforms that made it possible to share data on a wider scale. From there, they could proceed in one of two directions: either try and limit control to a few who were allowed to access the data, or to give wide access rights and trust the people to make good decision. However, there is a need for understanding what data can be shared and what cannot:

You are going to democratize your data, but you really can’t do that if you don’t understand what data you can democratize. Are people really allowed to use it? (CDO, Fiano)

By democratizing the data, the idea is to let everyone decide for themselves whether the data will give them the information they need or not. This can lead to changes in the principles for access management. Some informants gave access based on skills:

The data itself is managed within our multi cloud solution. From a data perspective the cloud platforms provide highly secure, massively scalable and agile delivery solutions. The cornerstone of our enterprise data and analytics platform, has been the establishment of our advanced cloud-based data science laboratory which provides the analytics capability and the technical scalability to over 40 data scientists across the organization. (CDAO, Barbera)

As pointed out, here only the data scientists had access to the data. Some of the organizations told us that they were aiming to build data products, that is, refined data sets combined with an application that processed the data and generated results, made easily available to a larger part of the organization. This because the competence needed to extract and use the data was an obstacle. While the earlier data storages was somewhat easier to extract information from, for instance data warehouses and buy business intelligence (BI) solutions, for the cloud-based data platforms of today, one needs algorithm and ML competence to extract and create a useful presentation of the data. Because such competence was hard to find, one of the organizations upskilled their employees:

What we saw was that we needed more data scientists, and it is hard to find people to employ. So, then we established a reskill program. There, we educated approximately 30 people over a year as data scientists. This was done through a cooperation with universities and through practical cases and work. (Senior VP, Barbera)

In addition to such upskilling, some of the informants also emphasized the importance of understanding the data and what you could actually achieve from using the data, as well as the possibility of supporting the learning process with data:

I believe that the area where we will see development now is that we will become better at using available data sources. We will develop a better gut-feeling on what data that actually informs us, and what is just nonsense. (Head of Innovation, Egiodola)

## Discussion

Our research question is: *how may data be gathered and used for information when organizations strive to become data-driven?*

When becoming data-driven, an early step is to understand what data is available and what data will need to be recorded. This may be data that was previously documented as information for record, that is, where the data was registered without a direct use. Such data may be valuable now, but the way of recording the data may not have been updated to a digital medium, as in the example of Jampal where some of the information was recorded in binders. We believe this to be a sign of a low-learning organization, and to change this behavior one would need to facilitate changes in the local work process. To provide the organization with data literacy competence as suggested by Berndtsson and Svahn (2020), may help somewhat, but more important would be to help the organization with redesigning the structure and process so that the data that is recorded provides meaning for it and opens it up for learning. The Barbera example, in contrast, showed an organization that is very engaged with its data and oriented towards making it more easily available to enable learning. In a STSD perspective we would expect that the processes that produce the data are designed in a way so that the recording of the data benefits the group that does the recording, for instance, by becoming their information for action. This may be done by creating their own interfaces, in a way consistent with the Archipelago architecture described by (Govers and van Amelsvoort 2018).

With regards to information for record we also saw that in some of the organizations there was individual ownership of the data and an unwillingness to share. As described in Gieryn (1983), the individual’s behavior may be intended to protect the worker’s professional field, and as Wulff and Finnestrand (2022) illustrated, this may well be because there is no alignment process that supports a more collaborative work environment. It may also be seen as a power issue—to have information is to have power—and if this is the case we would expect to find structures in the organization that promotes such behavior. For instance, that the quick clay experts are acknowledged for being sought after, that is, which creates a competition around who is the best expert. To alleviate such problems, the experts could be reorganized into an organizational structure with a common purpose to improve the detection of quick clay. This could help the transition from locally recorded data to making the data available for the collective, and to ensure that the data is made available and that it is used by the people needing the data to improve the way they work.

That data is recorded by people may become less common because many processes are digitalized and thereby provide data automatically. In addition sensors and other hardware are providing data. Nevertheless, the decision on what to record lies with the people designing the digital solutions, and to do so demands a knowledge of the work done that can only come from participation of the workers that perform the job. For instance, that the people doing the maintenance work are involved in gathering the data to find improvements.

Although recorded data may not be used for action in the organizational unit that records it, it is often useful for another business area, another organization, or perhaps for future use. The Senior VP from Barbera explained how the data quality is created in another part of the value chain than the one using it. This means that a part of data quality lies in organizational design. When the use of the data is in another area of the organization—or even outside of it—it challenges the idea of information for action being steered back to the group doing the action so that they can evaluate their actions and find new and better ways to achieve the intended outcome (Herbst 1974). This means that the organization can try to find mechanisms that will allow the group recording the data enough insight into the data quality the other group needs for their information for action. Perhaps there could be some kind of transparent feedback loop between the group recording the data and the one needing it for their action. This may create pride in the usefulness of the data. It also means that the exploration on what data to record should have wide boundaries.

The intention behind becoming more data-driven is to improve the efficiency of the work processes either through augmentation (supporting the worker) or automatization, to improve the customer experience, create new business models and to better support decision-making (Benbya et al. 2020). When it comes to information for action, it is supposed to aid the team doing the work. When the task given is to improve and automatize the work, the necessary competences needed may be so different from the competence to perform the work that other experts are invited in, for instance programmers. This creates a distance between the people performing the work as it is today and the people changing the work that should be kept as small as possible (Orlikowski 1992).

When the delivery is fully digital, as is the case for many of the processes in finance, the design and development team is doing the work, and can, with feedback try to continuously improve the deliveries. This means that much of the information for action when becoming data-driven is design-related. And the design includes the output of the team. One of the most notable differences between the work teams in British coal mines, which have informed most sociotechnical design studies (Trist and Bamforth 1951), and digital product development teams nowadays, is probably that coal miners were never encouraged to decide for themselves whether they should go looking for other products. They could not one day decide to go looking for gold, or emeralds, or to find out if it was possible to extract valuable minerals from the coal. Digital product development teams—like many other teams today—are constantly asked to create new products or services. As a result, information for action is not only necessary for employees in order to perform daily operations, but also in order to be able to be innovative and discover new products or services. Then, information for action is seen as a way to try to better understand what is going on in and around the organization, to be a learning organization (Herbst 1974, 1993). The difference between an organization that has a limited interest in learning from the data and an organization that is well underway to benefitting from its data have been described in the previous chapter. We would like to see even more organizational design research on how to help the low-learning organizations develop.

We found that the use of data to inform action is happening in the primary processes as well as in tactical and strategic processes, which aligns with the findings of Halper and Stodder (2017). This means that when designing the information that will lead to action there is a need to reflect upon all types of use, not only the use taking place in primary processes. What we also found was that data can be used for other purposes than intended, which means that there is a need for openness in the access to the data.

Cherns (1976, 1987) addressed work designers of the work in his seminal articles, but now the possibilities may also increase for the workers to design their information for action for themselves. The quality of work as described by de Sitter et al. (1997) implies that the workers are given sufficient autonomy to handle the variance they experience. As the variance they experience increases, the need for information to understand what is going on will probably increase as well. This is why one element of data sharing, the possibility to enable your own information through design might be useful. The new data platforms provide possibilities for using data in new and innovative ways. We saw that one of the experts from Fiano believed in the democratization of data, which means that people should be able to create their own information for action. However, this also affects design choices. Some of the interviewees believed that the access to the data should be restricted. This may be because they want to limit perceived vulnerabilities that appear if everyone can access data and build their own reports on what is going on. Or, because data access previously may have been restricted to the users of one particular IT-solution, this may be the way the access management has been defined. Now, there can be discussions on who will have access and when. This is why Davenport and Kirby (2016) explain that data analysts should make an effort to become data scientists, that is, programming the reports into ML models instead of using a lot of time to gather data and run analyses.

As demonstrated by Mitki et al. (2019), providing the workers in the organization with information on how the organization is performing is a powerful improvement tool. We believe such a sharing also to be part of the idea in the democratization projects performed by Emery and Thorsrud (1976), although then only in the business unit they were a part of. However, in some cases information is not used for action but for extraneous reasons, such as curiosity, which is why banks monitor who accesses a customer account as instructed by law. Such extraneous uses may be used to justify why the data access should be limited, however, we believe that the information for action needs should take precedence.

When it comes to improving the decision-making in the organization, STSD is clear on the principle that control is built bottom-up. This is why information for action is steered to the group/team doing the work. This means that the design principles for information for action for strategic purposes, needed for instance by of a board of directors or by top management, are difficult to deduce. As the possibilities for information for control increases with AI, we would like to encourage research into this area to provide examples or guidelines of what constitutes strategic information for action and what can be considered information for control.

## Conclusion and considerations for practice

Our study of 13 different organizations at different stages in their quest to become data-driven points to 4 findings to guide us.

First, when there is a need to record data that is not directly used as information for action by the people doing the recording, there may be challenges with regards to how well the data is gathered. We suggest that more research is done to find ways of handling this, for instance by making the actions supported by the data data transparent. To be able to utilize the opportunities that lie in being a data-driven organization, it is important to gather and systematize the information in such a way that it becomes *joint information for action*.

Secondly, for organizations where part or all of the delivery is digital, the information for action is often design-related. That is, it is used to redesign the digital processes. There is a danger that the development team will wish to avoid going into organizational development issues where only parts of the process are digital, like in the Jampal example. This is similar to what was described by Smith and Eckroth (2017), whose suggestion is to avoid affecting existing work process when designing AI solutions. To design such AI solutions can lead to investments into AI diverting from what is most beneficial to the organizations. Therefore, there is a need to understand what task structures can and should be redesigned and where AI can contribute.

Third, and building on this, becoming data-driven while still maintaining a flexible organization (Brödner and Latniak 2003) means that the information that is important for action today may not be what is important tomorrow. To ensure that the digital transformation results in a more flexible organization, we think it is essential to enable more people in the organization to participate in deciding what data to record in order for them to come up with new information and new uses for it. This means that people without prior knowledge of designing information for action are now expected to do this. To support their advances into building information for action, the data must not only be available, but the person must also have the required competence to build the information in compliance with the regulations.

Fourth, the need for information for action is spread across the whole organization. This means that it will differ where and what information is needed first, and that it is important to share it for the right purpose regardless of the person’s position. The information for action is, in addition to its use for operational action, also used to understand what is happening in the outside world. It is needed on several levels in the organization, by staff in operations, in design, and in leadership positions. This means that the boundaries of what can be categorized as information for action should be explored and reflected upon by the people involved in the redesign.

Our suggestion for future research is into the area of data design and organizing. We believe that now, perhaps more than ever, as the foundations for tomorrow’s data-driven businesses are being laid, there is a need for a strong presence from STS researchers and consultants. Therefore, it is important to engage in reflections on what data to record in order to build quality organizations. Not every data element that is produced can and should be stored, and the discussion on what data to use for action may inform the discussions about digitalization of the work.

Finally, becoming data-driven is sociotechnical in nature, and the major difficulties are nontechnical (Halper and Stodder 2017). Organizations are now hiring—or trying to hire—data scientists to handle their data and to seek value from it (Benbya et al. 2020). In addition we would encourage that they train or hire organizational designers. As we have pointed out, there are areas where there is benefits in upskilling or reskilling employees, and it could be beneficial to increase workers’ design knowledge (Parker and Grote 2022) so that they are able to design and use the data for action.

At the same time that we interviewed the experts, another software consultancy in Norway made a quantitative survey of the situation in Norway with regards to becoming data-driven. One of their findings was formulated as *spreadsheets are (still) king *(Almquist et al. 2021, p. 7) which points to the same individual use of data that we found. They also point out that where leaders have a clear accountability for making data available, it is democratized. This means that the need for joint information for action still waits to be discovered in some organizations, and that leaders will need to both make the data available and see to it that the people in the organization can and will use it.

Summing up, the short answer to our research question of how data is gathered and used when becoming more data-driven is: not easily. As we have illustrated there are organizational design roadblocks to handle, for instance where the organizational design has not supported mutual learning so far, or where the process is divided so that the people expected to gather the data are not the ones reaping the benefits. Then again, we have seen examples of organizations that have managed to provide their employees with the room and the competence needed to progress the organization. This, hopefully, will provide inspiration to others trying to become more data-driven.
